# Analysis and intelligent prediction of domino effect accidents in chemical storage tanks with a focus on accident chain length

**DOI:** 10.1371/journal.pone.0331180

**Published:** 2025-09-02

**Authors:** Jinrong Qi, Mingguang Zhang, Guo Yu, Cuimei Bo

**Affiliations:** 1 College of Safety Science and Engineering, Nanjing Tech University, Nanjing Jiangsu, China; 2 Jiangsu Key Laboratory of Hazardous Chemicals Safety and Control, Nanjing Jiangsu, China; 3 Institute of Intelligent Manufacturing, Nanjing Tech University, Nanjing, Jiangsu, China; 4 College of Electrical Engineering and Control Science, Nanjing Tech University, Nanjing, Jiangsu, China; Southwest Petroleum University, CHINA

## Abstract

The compact arrangement of chemical storage tanks significantly increases the occurrence probability of domino effect accidents. The accident chain length, a critical parameter for assessing accident severity, enables rapid comprehension of potential accident impacts and serves as a foundation for constructing accident scenarios in domino effect risk assessment. This study centers on domino effect accidents within chemical storage tanks and conducting a detailed analysis of factors influencing the accident chain length. Given the limitations in historical statistical data and quantitative risk evaluations, an intelligent prediction method is developed to forecast the accident chain length. A fully connected feedforward neural network (FC-FNN) is utilized to analyze 255 pertinent accident cases spanning from 1970 to 2024, with key features such as the type of substances implicated and the operating conditions during accidents being judiciously screened. To compensate for the insufficiency of data regarding the volume of storage tanks, a small-scale augmentation is implemented within the tolerable error range. Additionally, Shapley Additive Explanations (SHAP) is applied to optimize the feature set, reducing the number of features from 15 to 10 based on their contribution to the model’s predictions. The results show that the combined application of feature selection, data augmentation, and SHAP-based optimization significantly improves the model’s prediction performance. The test set prediction accuracy exceeds 0.978, demonstrating the effectiveness of the proposed approach.

## 1. Introduction

Benefiting from significant improvement in scale economy effects, industrial chain integration, and environmental advantages, chemical industry parks (CIPs) have emerged rapidly worldwide [1]. However, this development pattern has introduced safety challenges, as high-density clustering of chemical plants and the substantial hazards they pose have undeniably increased the potential risks of safety accidents, such as fires and explosions [1,2]. In the event of an accident, its impact can rapidly propagate to adjacent plants or enterprises, triggering a domino effect that results in a series of accidents [3]. Chemical storage tanks, as critical vulnerability points of the chemical industry, often store flammable and explosive substances, making them susceptible to accident propagation throughout industrial complexes [3,4].

Reviewing historical accidents involving hazardous chemicals, it becomes evident that domino effect accidents are quite prevalent [3]. Accident chains, as crucial factors in identifying domino effect accidents, are essential for predicting accident trends and guiding accident prevention and control measures [5]. Text mining can be utilized to extract accident chains, thereby enhancing the accuracy of accident identification and prediction [6].To elucidate the characteristics of domino effects, some scholars have conducted statistical analyses on domino accidents. However, such research is limited, primarily due to the insufficient number of accident cases for statistical purposes or incomplete accident information, making it difficult to ascertain whether a domino effect is involved [7]. Darbra et al. collected 225 domino effect accidents and summarized the areas most prone to domino effects and the most frequent accident sequences [8]. Hemmatian B et al. analyzed 330 historical accidents and compared them with Darbra’s statistical results, finding that production areas (38.5%) and storage tank areas (33%) remained the most common locations for accidents, with a ratio of 6 between primary and secondary extended accidents [9]. Guohua Chen summarized the statistical analysis results of numerous scholars on accident cases and discovered that secondary accidents are the most numerous in domino effect accident chains [10]. In “two-step” domino effect accidents, the most significant types are “fire→explosion” and “explosion→fire”. In “three-step” domino effect accidents, the most important types are “fire→explosion→fire” and “explosion→fire→explosion”. Zhang et al. analyzed 165 domino effect accidents worldwide and extracted key accident nodes as “fire→fire”, “fire→explosion”, “explosion→fire”, and “explosion→explosion” [4]. Liang et al. conducted a statistical analysis of 49 domino effect accidents involving atmospheric storage tanks and found that tank explosion accidents accounted for 55.1% [11]. Despite these insights, the lack of granular data on materials and equipment limits the applicability of statistical results. This gap has spurred interest in dynamic models capable of predicting, rather than merely describing, accident propagation [12–15].

In view of the limitations of traditional research in capturing the characteristics of domino – effect accidents and their practical applications, as well as the pressing need for constructing dynamic accident models, the increasing complexity of industrial systems in recent years has led to the emergence of research on system resilience within the field of domino effect studies [16,17]. The resilience theory emphasizes that when confronted with disturbances and shocks, a system can not only maintain its basic functions but also recover rapidly and achieve adaptive adjustments [18]. The introduction of this theory aims to address the deficiencies in traditional research regarding the dynamic evolution of accidents and the analysis of system response capabilities. In the context of chemical industrial parks, system resilience is closely related to the prevention and control of domino effects. A highly resilient system can effectively prevent the spread of domino effects and reduce the extension of accident chains when accidents occur [17,19]. This echoes the research on domino – effect accident chains and provides new ideas for controlling accident development and reducing accident losses. In recent years, some scholars have, from the perspective of system resilience, attempted to enhance the ability of chemical industrial parks to withstand domino – effect impacts by means of optimizing park layout, strengthening equipment redundancy design, and establishing dynamic risk early – warning mechanisms [20]. These practical explorations essentially aim to seek more forward – looking and proactive risk prevention and control strategies based on existing accident models and statistical analyses. While resilience theory provides a conceptual framework, its quantitative application to domino effects remains underdeveloped, necessitating data-driven approaches like machine learning [21]. This further highlights the necessity of this study to explore new methods and develop more effective prediction models.

Conventional statistical methods are inadequate for characterizing domino effects and fail to satisfy the stringent requirements of modern chemical process safety. Domino accidents in industrial settings typically cause exponentially greater losses than isolated incidents. Consequently, significant research efforts focus on domino effect risk identification, assessment, and management [22–29]. All of these efforts involve the identification and construction of Domino Effect accident scenarios. The formulation of Domino Effect emergency response plans and the dispatch of emergency resources rely on an accurate grasp of the accident scenarios [30–32]. The chain length of Domino Effect accidents, a parameter influenced by multiple factors such as the types of substances involved, the accident area, and the accident’s propagation mode, can effectively reflect the overall severity of the accident. It provides a basis for identifying key nodes in accident scenario construction and serves Domino Effect risk assessment and emergency management.

With the development of machine learning theories, computer hardware, and programming languages, the application of machine learning in the chemical industry has become increasingly widespread, to extract information, identify patterns, and make predictions [33]. Many scholars have combined data with deep learning algorithms to conduct predictions on target objects, achieving relatively high prediction accuracy and resolving the issue that traditional static data has difficulty in accommodating the dynamic changes of data [34–36]. Currently, using historical data to predict the development trend of accidents has become a research hotspot. The fully connected feedforward neural network demonstrates distinct advantages in terms of prediction accuracy, generalization ability, etc., effectively making up for the deficiencies of traditional empirical models [37–39]. The statistical data of chemical storage tank accidents are characterized by a large number of technical terms and strong data correlations, and the fully connected architecture of FC-FNN can precisely match these characteristics. When dealing with complex textual data, the word embedding technique can better capture semantic information and provide the hidden semantic relationships between words [40–42]. SHapley Additive exPlanations (SHAP), when combined with machine learning models, can quantify the contribution of each feature to model predictions. As a result, SHAP values have emerged as a key metric for evaluating feature importance in predictive models, enabling feature optimization and enhancing model interpretability [43,44]. Given the multifaceted challenges in domino effect accident research and the urgency for precise prediction and efficient management, along with the distinctive merits of advanced technologies like the fully connected feedforward neural network and SHAP values, this study aims to utilize these techniques comprehensively. It will deeply analyze the chain length of domino effect accidents and build a more accurate and effective prediction model, expecting to strongly support the risk assessment, emergency management, and accident scenario construction of such accidents, thereby better addressing this critical practical issue.

Specifically, an exhaustive collection of the data regarding chemical storage tank domino accidents since 2024 was carried out, and a fully connected feedforward neural network (FC-FNN) [45], employing word embedding for text classification was constructed to intelligently prognosticate the chain length of the domino effect accident chain in chemical storage tanks. In the feature selection phase, a statistical analysis of 255 historical accidents was performed for feature dissection, and the crucial influencing factors related to chain length were chosen as feature inputs. To verify the rationality of feature selection, SHAP values were introduced. By quantifying the contribution of each feature to the model’s prediction results, the feature selection process was further optimized. Moreover, data augmentation techniques were applied to guarantee the precision of the prediction model, thereby providing a novel approach for the construction of accident scenarios for this category of accidents.

The subsequent structure of this paper is as follows: Section 2 elaborates in detail on the basic concepts of domino effect accidents and the length of their accident chains, and also introduces that we have introduced a feedforward neural network based on word embedding for the statistical characteristics of domino effect accident data. Moreover, to identify highly relevant features, we introduce the SHAP theory. Section 3 explains the algorithm we designed and its implementation process. Section 4 clarifies the analysis results of domino effect accidents in chemical storage tanks by comparing with existing literature and discusses the experimental results of the intelligent prediction of the length of their accident chains. The conclusion is drawn in Section 5.

## 2. Related work

Firstly, this section defines domino effect accident and the chain length. Then, a Word Embedding-based FC-FNN tailored to the statistical characteristics of domino effect accident data is introduced. Finally, the SHAP theory is incorporated to enhance the model’s interpretability and conduct feature importance analysis. These steps establish the theoretical framework for understanding the predictive modeling approach of domino effects and the interpretability framework subsequently employed in this study.

### 2.1. Definition of domino effect accident

As a high-impact low-probability (HILP) accident scenario, domino effect accidents have garnered growing public concern [46]. Characterized by their inherent complexity, precisely defining these accidents poses a formidable challenge [47]. To date, the academic community has yet to reach a consensus on a standardized definition. Although there is much controversy, in the chemical industry, the term “domino effect” refers to a series of accidents, in which the main accident is usually a fire or explosion, which triggers further accidents and comprehensively escalates the consequences of the event [48].

Currently, the most widely – accepted definition of ‘domino effect accident’ stems from the description of the requisite conditions for the existence of the domino effect put forward by Cozzani [49]: ①An initial accidental scenario exists, which initiates a domino accident; ②The physical expansion consequence of the initial event leads to the aggravation of the accident, meaning that the propagation of the initial accident must lead to the malfunction of at least one secondary equipment unit; ③At least one secondary accidental scenario occurs; ④There is an accident expansion effect, i.e., the overall severity of the domino accident surpasses that of the initial accident.

In the subsequent research of this paper, the following terminologies will be employed:

(1) Initial accident: The first accident triggered by accidental factors.
(2) Accident expansion: The process whereby an accident in one storage tank induces an accident in another adjacent storage tank. This process can be the transmission of the same type of accident or the transmission of different types of accidents.

### 2.2. Definition of chemical storage tank domino effect accident chain

According to the disaster system theory, disasters are the result of the interaction of hazard-inducing factors, disaster-pregnant environments, and disaster-bearing bodies [50]. In chemical industrial parks, hazard-inducing factors are primarily categorized into natural disaster – related and technological disaster – related factors. The latter includes domino effect escalation factors, namely fire heat radiation, explosion shock waves, and explosion fragments. In this study, the disaster-bearing body is the tank farm. During the expansion of the accident chain, it predominantly spreads through dangerous equipment units that can trigger fire and explosion incidents upon failure. The chain affects equipment, personnel, and the environment via hazard-inducing factors such as fire heat radiation, explosion shock waves, and explosion fragments. The disaster-pregnant environment encompasses the natural and humanistic environments within and outside the park, as well as the interrelationships among disaster-bearing bodies, including meteorological conditions, personnel distribution, management factors, and park layout. The severity of disaster consequences is jointly determined by the hazard of hazard-inducing factors and the vulnerability of disaster-bearing bodies.

In the evolution of a domino effect accident, the length of the accident chain is defined as the number of accident propagation events from the initial accident facility to the end of a series of subsequent chain reactions. The initial accident facility marks the starting point, while the final impacted facility denotes the endpoint of the accident chain. This length is determined by calculating the number of times the accident propagates between facilities, rather than simply counting the total number of affected facilities. For instance, if an accident spreads from Facility A to B, and then from B to C, the length of the accident chain is 2 (A → B → C, with two propagation events). When the accident spreads simultaneously from A to B and A to C, the chain length is 1 (since A → B and A → C are parallel propagation paths, with a single propagation event). In a scenario where the accident spreads from A to B, then from B to C, and concurrently from A to D, the chain length is 2, as the longest propagation path (A → B → C) involves two propagation events. The accident chain length is intricately linked to the hazard of hazard-inducing factors and the vulnerability of the affected carrier. Consequently, it is influenced by multiple factors, including the types of substances involved, the accident location, and the mode of accident expansion. The accident chain length serves as an effective indicator of the severity of such disaster consequences. It facilitates the quantitative risk assessment of such accidents by pinpointing risk sources and high-risk areas for scenario construction, and also aids in identifying the origin and critical links of accidents.

### 2.3. Word embedding-based feedforward neural network

Accurate intelligent prediction of domino effect accidents in chemical storage tanks necessitates statistical analysis of existing relevant cases. Since the statistical items are numerous and all key information extracted from textual data, we elected to establish a fully-connected feedforward neural network based on word embeddings for text classification. This neural network architecture exhibits robust representational learning capabilities. It can transform input data through layered linear and nonlinear operations, gradually converting them into more abstract and hierarchical feature representations. By doing so, it effectively captures the inherent structures and patterns within the input data, thereby significantly enhancing the prediction accuracy for domino effect accidents in chemical storage tanks.

To process textual data effectively, this paper utilizes Word2Vec [51] embeddings to transform the selected features from text data into vectors. This approach encodes the semantic relationships between words, providing meaningful inputs for the neural network and enabling efficient feature extraction and classification. Each word embedding serves as a feature input, which undergoes nonlinear transformation in the hidden layers using the Rectified Linear Unit (ReLU) activation function. Neurons in each layer are connected to the previous layer through weights, facilitating the gradual extraction and representation of abstract features within the input text. At the output layer, the softmax function calculates the probability distribution across different categories, determining the likelihood of the text belonging to each class.

The fully connected layer plays a crucial role in performing both linear and nonlinear transformations on the input data for feature extraction and transformation. Its implementation is described by equations 1.1 and 1.2.


z=W·x+b (1.1)

Wherein, z represents the output of the linear transformation, which is the weighted sum of the inputs plus a bias term. W is the weight matrix, where each element represents the weight of a connection between an input feature and a neuron. x is the input vector to the layer, with each element representing an input feature. b is the bias term, added to the weighted sum to allow the model to better fit the data by shifting the activation function.


y=ReLU(z) (1.2)

Wherein, y is the output of the ReLU activation function. ReLU(z) represents the Rectified Linear Unit activation function, which is defined as ReLU(z)=max(0,z). It introduces non-linearity to the model by outputting the input directly if it is positive; otherwise, it outputs zero. z is the input to the ReLU function, which is the output from the linear transformation in equation 1.1. During the training process of a neural network, Dropout is employed to randomly discard a portion of neurons, enabling the network to learn more robust and generalized features while reducing co-adaptation between neurons. equation 1.3 specifically represents the implementation of the Dropout layer.


$$y=\frac{1}{1-\text{ƞ}}\times x\times \text{mask}$$ (1.3)

Wherein, x is the input to the Dropout layer, y is the output from the Dropout layer, η is the dropout probability, mask and x is a randomly generated binary mask of the same shape as, where 0 indicates a neuron that is dropped out, and 1 indicates a neuron that is retained.

### 2.4. SHAP value theory

To improve model interpretability, we incorporated SHAP values for feature selection. Rooted in Shapley values from game theory, SHAP values enable a fair apportionment of each feature’s contribution to the model’s prediction outcomes. We employed SHAP values to analyze the feature importance of the Word Embedding-based Feedforward Neural Network and selected the most influential features for model retraining.

For the model prediction result f(x),the SHAP value $\phi_{i}$ is defined as:


$$\begin{array}{c} \phi_{i}=\sum {\mathrm{\backslash nolimits}}_{S\subseteq F\{i\}}\frac{|\text{S}|!(|\text{F}|-|\text{S}|-\text{1})!}{|F|!}(f\left(S\cup \left\{i\right\}\right)-f\left(S)\right) \end{array}$$ (1.4)

Where F is the set of all features, S is a feature subset, and f(S) is the model prediction result based on the feature subset S.

## 3. Proposed method

In this section, we will prepare for developing an intelligent prediction model for the chain length of domino effect accident chains in chemical storage tanks by collecting the historical accident data of the domino effect in chemical storage tanks and analyzing to identify the influencing factors related to the length of the accident chain. In the establishment of the model, we will first outline the proposed algorithm framework and then elaborate on the aspects of feature selection and data augmentation in detail.

### 3.1. Framework

We collected data on domino effect accidents involving atmospheric and pressurized storage tanks from 1970 to 2024, both in China and internationally. These data were then summarized and statistically analyzed. Among the evolutionary characteristics of domino effect accidents, the chain length of the accident chain is a critical parameter used to characterize the overall severity of the accident. To conduct a comprehensive analysis, we consider a wide range of accident statistical characteristics, including but not limited to: the category of the initial accident device, the name of the initial accident device, the form of the storage tank, the tank capacity, the tank material, the initial accident medium, the pressure state, the type of initial medium, the type of initial accident, the secondary accident unit, the chain length of the accident, the status of the unit at the time of the accident, and the location of the initial accident.

The framework of this study is structured as follows:

1. **Data Preprocessing**: The raw data is preprocessed through feature selection and data augmentation to ensure its suitability for modeling.
2. **Feature Embedding**: The preprocessed text data is converted into vector representations using the Word2Vec embedding technique, enabling the use of machine learning models.
3. **Chain Length Prediction**: A fully connected feedforward neural network is trained on the embedded data to predict the chain length of domino effect accidents.
4. **Feature Optimization with SHAP**: Identify the most influential features and enhance the interpretability of the model.

Algorithm 1. Intelligent Prediction Model for Domino Effect Accident Chain Length

Data collection and analysis of Domino Effect Accidents in Chemical Storage Tanks

Input: Summary Data of Domino Effect Accidents

//Feature Selection:

1: Extract Input Features & Corresponding Chain Lengths

//Data Augmentation:

1: if Tank Capacity Present

2: Extract Unit + Value

3:Numeric Value× random (0.99 ~ 1.01);

4: end if Blank Tank Capacity

5: Duplicate this row of data;

//Model Training and Prediction:

1:Feature Matrix→Word2Vec→Numeric Vector

2:Numeric Vector+Corresponding Chain Length→Feedforward Neural Network

//SHAP-based Feature Optimization:

1: Apply SHAP to the trained FC-FNN model

2: Calculate Shapley values for each feature to quantify its contribution to the model’s predictions

3: Rank features based on SHAP values to identify the most influential ones

4: Refine the feature set by retaining only the top influential features

5: Retrain FC-FNN with the optimized feature set for improved prediction accuracy

Output: Chain Length and Feature Importance Analysis

### 3.2. Data collection and analysis of Domino Effect Accidents in Chemical Storage Tanks

This paper collects 255 domino effect accident cases involving atmospheric and pressurized storage tanks and their auxiliary pipelines, sourced from Chinese and international petrochemical enterprises spanning 1970–2024 (with all cases recorded through the end of 2024). These cases serve as analytical samples to characterize tank-related domino accidents. Specifically, 143 cases are identified as atmospheric storage tank domino accidents, and 95 as pressurized storage tank domino accidents.

**Fig 1:**
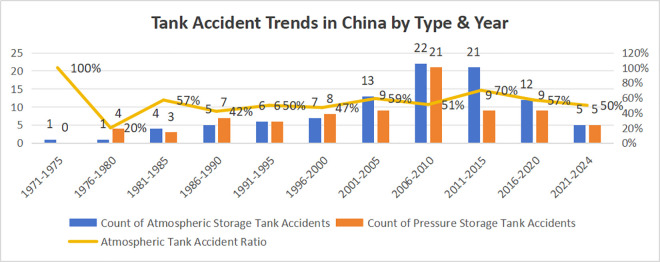
Development trend of chemical storage tank accidents in China (1970–2024, including data from 2024).

To demonstrate the development trend of the number of storage tank accidents in China, a five-year period is adopted for analyzing the quantitative trend of domino accidents involving both atmospheric and pressurized storage tanks. As shown in Fig 1, the number of accidents generally exhibits a trend of first increasing and then decreasing. Specifically, it shows an upward trend from 1970 to 2010 and begins to decline after 2010. In terms of the proportion of domino accidents involving atmospheric storage tanks, the number of such accidents accounts for more than 50% of the total.

#### 3.2.1. Substance type analysis.

Table 1 summarizes 143 domino accidents involving 31 types of hazardous materials in atmospheric storage tanks. Among liquid substances, gasoline (21.7%) accounts for the highest proportion of causing atmospheric storage tank accidents. Among explosive volatile gases, oil evaporation gas (18.8%), hydrocarbon-containing evaporation gas (18.8%), and benzene-containing evaporation gas (18.8%) are the top three contributors to atmospheric storage tank accidents.

**Table 1:** Initial material causing atmospheric tank domino accident in China and internationally.

Initial Accident Material	Accident Count	Percentage %	Initial Accident Material	Accident Count	Percentage %
Gasoline	31	21.7	Heavy Oil	2	1.4
Crude Oil	22	15.4	Hydrogen Peroxide	2	1.4
Explosive Volatile Gases	16	11.2	Kerosene for Lamps	1	0.7
Diesel Fuel	9	6.3	Carbon Disulfide	1	0.7
Naphtha	9	6.3	Aromatic Hydrocarbons	1	0.7
Fuel Oil	5	3.5	Blended Oil Products	1	0.7
Contaminated Oil	5	3.5	Oil-Based Cleaning Solution	1	0.7
Toluene	4	2.8	Iron Sulfide	1	0.7
Alkanes	4	2.8	Light Oil	1	0.7
Benzene	3	2.1	Nitrotoluene	1	0.7
Heat Transfer Oil	3	2.1	Isopropanol	1	0.7
Methanol	3	2.1	Liquid Wax	1	0.7
Wax Oil	3	2.1	Methyl Tertiary Butyl Ether	1	0.7
Residue Oil	3	2.1	Chloramine	1	0.7
Coal Tar	3	2.1	High-Boiling Slurry	1	0.7
Solvent Oil	2	1.4	—	—	—

Table 2 summarizes 95 initial material accidents in pressurized storage tank areas, involving 15 types of substances, which are categorized into liquefied gases (60%) and compressed gases (40%). Among liquefied gases, liquefied petroleum gas (LPG) accounts for the highest probability as the initial material, at 29.5%. Within compressed gases, explosive mixed gases (combinations of CH4, H2, etc.) have the highest probability of being the initial material, at 14.7%.

**Table 2:** Initial material causing pressure storage tank domino accident in China and internationally.

Initial Accident Material	Accident Count	Percentage %	Initial Accident Material	Accident Count	Percentage %
Liquefied Petroleum Gas	28	29.5	Ethylene Oxide	3	3.16
Explosive Mixture Gas	14	14.7	Ammonia Gas	3	3.16
Liquefied Natural Gas	12	12.6	Liquid Oxygen	3	3.16
Liquid Ammonia	7	7.37	Butane	2	2.11
Hydrogen Gas	6	6.32	Propane	2	2.11
Liquid Chlorine	5	5.26	Natural Gas	2	2.11
Ethylene	3	3.16	Chlorine Gas	2	2.11
Toxic Gas	3	3.16	—	—	—

#### 3.2.2. Operational status during accident occurrence and cause analysis.

Studying the operational status during accident occurrence is highly effective in identifying high-risk operations and reducing the probability of accidents. According to statistical findings, the probability of accidents occurring under normal working conditions is 40.4% for atmospheric storage tanks, while it is 15.4% during inspection and maintenance. For pressurized storage tanks, the probability of accidents occurring under normal working conditions is 56%, and 19% during inspection and maintenance.

The analysis of accident causes is crucial for the prevention of domino accidents. Based on the accident causation theory of chain reaction and incorporating the categories of accident causes described in MHIDAS, a classification analysis of the contributing factors to the accidents under study has been conducted. Human factors are identified as the primary cause of accidents (50%), followed by equipment factors (27.9%) and environmental factors (24.3%). Among human factors, violation of work regulations (20.6%) accounts for the highest proportion, closely followed by improper operation (19.9%).

#### 3.2.3. Abbreviated analysis of accident chain evolution & propagation.

The most prevalent accident chain in atmospheric storage tanks involves gasoline, with the common modes being “Leak-Explosion-Fire→Fire” and “Leak-Fire→Fire”. Crude oil follows, primarily causing “Leak-Fire→Fire”. Explosive volatile gases often lead to “Explosion→Fire”. Leakage is the most frequent initial event triggering domino effects in atmospheric tank farms. As shown in Table 3, the most common spread mode among adjacent tanks is “Fire→Fire” (59, 39.9%), followed by “Explosion→ Fire” (36, 24.3%) and “Fire→Explosion” (35, 23.6%).

**Table 3:** Escalation mode of atmospheric tank accident chain.

Propagation Mode	Number of Accident Chain Segments	Probability
Fire → Fire	59	0.399
Explosion → Fire	36	0.243
Fire → Explosion	35	0.236
Fire → Leakage	5	0.034
Explosion → Explosion	4	0.027
Explosion → Leakage	4	0.027
Leakage → Fire	3	0.020
Leakage → Explosion	2	0.014

Given that the propagation of accident chains in tank farms is not endless, when human control measures are implemented, the accident chain will terminate at a certain point without affecting all tanks in the tank farm. To study the transmission length of accident chains, the average chain length of accidents is defined as the sum of the transmission lengths of all accident chains divided by the number of accidents. Here, the length of an accident chain refers to the number of times an accident expands; if an accident expands once, the length of the accident chain is 1. According to the analysis in Table 4, the average chain length of the 143 accidents is 1.17. This indicates that, in most cases, the domino effect of accidents in atmospheric storage tank farms terminates after affecting two tanks due to human emergency intervention. In a minority of cases, initial emergency response delays can lead to the expansion of the domino effect, triggering accidents in multiple tanks in the tank farm. The analysis of the average accident chain length of the domino effect in tank farms provides basic data for the construction of accident chain evolution and expansion scenarios in atmospheric tank farms.

**Table 4:** Statistics of the accident chain length in the atmospheric tank farm.

Number of Expansions	First-order Expansion	Second-order Expansion	Third-order or Higher-order Expansion
Number of Accidents	120	21	2

In pressure tank accidents, leaks account for the highest proportion (80%) of initial incidents. Among these, secondary first-order accidents occur at a rate of 30.5%, with “Leak-Explosion” being the most frequent chain. For secondary second-order accidents (48.4%), the most prevalent chain is “Leak-Explosion-Fire”. As for secondary third-order accidents (21.1%), the most common chain is “Leak-Dispersion-Explosion-Fire”. As shown in Table 5, the most common propagation mode among adjacent tanks in pressure tank farms is “Explosion → Fire” (40, 34.8%), followed by “Leak → Explosion” (30, 26.1%) and “Dispersion → Explosion” (22, 19.1%).

**Table 5:** Escalation mode of pressure storage tank accident chain.

Propagation Mode	Number of Accident Chain Segments	Probability
Explosion → Fire	40	0.348
Leakage → Explosion	30	0.261
Dispersion → Explosion	22	0.191
Fire → Fire	7	0.061
Explosion → Explosion	6	0.052
Fire → Explosion	5	0.043
Leakage → Fire	5	0.043

Calculations based on Table 6 reveal that the average extension length of accident chains in pressure tank farms is 1.91, indicating that once an accident occurs in a pressure tank farm, it is highly likely to lead to secondary second-order accidents.

**Table 6:** Statistics of the accident chain length in the pressure tank farm.

Number of Expansions	First-order Expansion	Second-order Expansion	Third-order or Higher-order Expansion
Number of Accidents	29	46	20

### 3.3. Feature selection

In the feature selection stage, we first conducted an analysis based on domain knowledge and historical data, and selected 15 features that might be related to the length of the accident chain. These features include weather conditions, air temperature, alarm response speed, the category of the initial accident device, the name of the initial accident device (named in the form of “medium + storage tank; process + device; medium + device”), the form of the storage tank, the tank capacity, the tank material, the initial accident medium, whether it is under normal pressure or pressurized, the type of the initial medium, the type of the initial accident, the secondary accident device, the status of the device at the time of the accident, and the location of the initial accident. To verify the rationality of these features, SHAP values were used to calculate the contribution of each feature and conduct an assessment.

**Fig 2:**
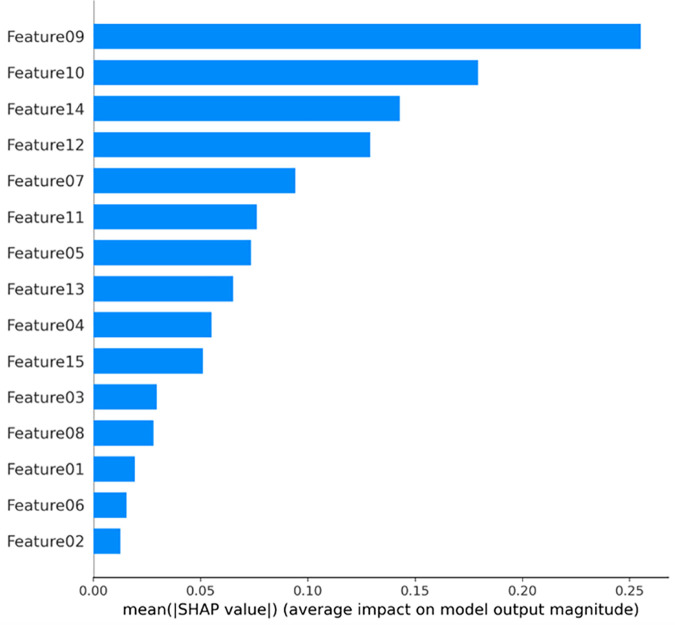
SHAP-based feature importance ranking.

The 15 identified features are sequentially labeled as Feature 01 to Feature 15, and SHAP is applied to evaluate their importance. Fig 2 presents an aggregated SHAP-based feature importance plot, highlighting the top 10 critical features for predicting accident chain length. Notably, the initial accident medium (Feature 09) exhibits the highest SHAP value, indicating its dominant role in propagating domino effects, which aligns with statistical findings that over 80% of domino accidents involve flammable/explosive substances. Second in importance is the storage tank pressure condition (Feature 10), as pressurized tanks are more prone to secondary accident escalation, leading to accident chains ≥2 in length. The equipment operational state during the accident (Feature 14) ranks third, with most incidents occurring under normal operating conditions, primarily due to human error (e.g., non-compliant operations). Subsequent key features include initial accident type (Feature 12), storage tank volume (Feature 07), initial medium classification (Feature 11), primary accident equipment identifier (Feature 05), secondary accident equipment (Feature 13), primary equipment category (Feature 04), and accident location (Feature 15).

SHAP values analysis reveals that certain features contribute very little to the prediction of accident chain length. For example, the alarm response speed and storage tank material have low correlations due to limited data availability in accident reports. Additionally, features such as weather conditions, storage tank types, and air temperature have low SHAP value contributions and are therefore excluded from the final feature set. In summary, we have selected 10 high-correlation features for subsequent model training and prediction.

### 3.4. Data augmentation

To mitigate the issue of limited data, we partition the dataset into two subsets based on whether the storage tank capacity feature is missing or not. For the subset with non-missing tank capacity data, data augmentation is performed to enhance the sample size. While this augmentation may alter the statistical occurrence probability of such accidents, it does not compromise the prediction accuracy of domino effect accident chain lengths involving chemical storage tanks. According to the literature in Spherical and Large-Scale Storage Tanks [52], there are four fundamental concepts of tank capacity: calculated capacity, nominal capacity, actual capacity (storage capacity), and operating capacity. The actual capacity (storage capacity) refers to the maximum volume that a tank can physically hold. In accident investigation reports, the tank capacity mentioned typically corresponds to the actual capacity, signifying the maximum liquid volume the tank is designed and specified to contain. This capacity is determined by the tank’s design and specifications. To augment the tank capacity feature, we consider the permissible dimensional errors specified in relevant standards. According to GB 50341 *Design Code for Vertical Cylindrical Steel Welded Storage Tanks* [53], the allowable manufacturing error for storage tank capacity is ± 1%. Therefore, data augmentation is performed on the tank capacity feature within the range of 0.99 to 1.01 times the original value, ensuring compliance with industrial standards while expanding the dataset. The augmented data is then incorporated into the training set of our neural network model to ensure prediction accuracy.

From a fairness perspective, we also perform a replication operation on the non-missing tank capacity data to address potential biases arising from data imbalance in machine learning. This approach ensures that the model is trained on a balanced dataset, thereby improving its generalization ability and reducing the risk of overfitting to the majority class.

## 4. Experimental results and analysis

### 4.1. Statistical feature result analysis

Through comparisons with historical literature, Table 7 reveals five distinct characteristics of domino accidents in chemical storage tanks: flammable/explosive substances like liquefied petroleum gas, gasoline, and crude oil remain the primary initial accident materials consistent with prior studies; tank farms (48%) and production areas (18%) emerge as high-risk zones aligning with broader chemical industry trends; dominant expansion sequences include “fire→explosion,” “explosion→fire,” and “fire→fire” showing no significant deviations from traditional accident statistics; while atmospheric storage tank accidents still primarily involve single expansions (78%), pressure storage tank incidents exhibit a notably higher rate of secondary/higher expansions (69.5%) surpassing benchmark values. Critically differing from historical datasets, leakage is identified as the preponderant initial event particularly in pressure storage tank accidents (80% incidence), highlighting unique risk profiles for pressurized systems.

**Table 7:** Comparison with the relevant statistical literature.

Source	2010	2014	This paper	
	Darbra	Hemmatian B		
Statistical Period	1961-2007	1961-2013	1970-2024	
Number of Accidents	225	330	255	
Domino Accident Type	Chemical Industry Accidents	Chemical Industry Accidents	Chemical Industry Atmospheric Storage Tank Accident	Chemical Industry Pressurized Storage Tank Accident
Vulnerable Substances	Liquefied Petroleum Gas (27%)	Liquefied Petroleum Gas(22%)	Gasoline (21.7%)	Liquefied Petroleum Gas(29.5%)
	Crude Oil (11%)	Gasoline (10%)	Crude Oil (15.4%)	Liquefied Natural Gas(12.6%)
	Gasoline (11%)	Crude Oil (9%)	Explosive Volatile Gases (11.2%)	Explosive Gas Mixture(14.7%)
	Naphtha (6%)			
Accident Causes	External Events (31%)	External Events (29.4%)	Environmental Factors (24.3%)	
	Mechanical Failures (29%)	Mechanical Failures (35.2%)	Mechanical Failures (27.9%)	
	Human Error (21%)	Human Error (24.6%)	Human Error (50%)	
Initial Accident Type	Explosion (35.5%)	Explosion (53%)	Explosion (38.5%)	Explosion (16.8%)
	Leakage (32%)	Fire (47%)	Leakage (45.5%)	Leakage (80%)
	Fire (32.5%)		Fire (16%)	Fire (3.2%)
	(Ignoring Leakage)			
	Fire (52.4%)			
	Explosion (47.6%)			
Accident Location	Storage Area (35%)	Production Area (38.5%)	Storage Facilities (47.8%)	
	Production Area (28%)	Storage Area (33%)	Production Facilities (17.4%)	
	Transportation Area (19%)	Atmospheric Storage Tanks (18.6%)		
Expansion Patterns	Fire→Explosion (27.5%)	Fire → Explosion (28.8%)	Fire → Fire (39.9%)	Explosio →Fire (34.8%)
	Explosion→Fire (27.6%)	Fire → Fire (15.7%)	Explosion → Fire (24.3%)	Leak→ Explosion (26.1%)
	Explosion → Fire-Explosion (9%)		Fire → Explosion (23.6%)	Diffusion → Explosion (19.1%)
1st-Level Expansion Accidents	193	282	120	29
2nd-Level and Above Expansion Accidents	32	48	23	66

### 4.2. Intelligent prediction result analysis

#### 4.2.1. Model performance evaluation: Accuracy metric.

The accuracy of the experimental data is obtained by comparing the accident chain length predicted by the model with the actual accident chain length. Specifically, we use historical accident data to train a feedforward neural network model. On the test set, the accident chain length predicted by the model is compared with the actual accident chain length, and then the accuracy is calculated. The actual accident chain length is extracted from the historical accident data. In this way, we are able to evaluate the performance of the model under different data processing methods.

#### 4.2.2. Comparative analysis of data processing methods.

Table 8 presents the simulation results of training the neural network using the original data, data with added feature selection, data with augmented data, data with both added feature selection and data augmentation, and data with SHAP-based feature optimization.

**Table 8:** Simulation results of each part of the data.

Data Type	Augmentation Amount	Replication Amount	Training Set Accuracy	Test Set Accuracy
Original Data	0	0	0.40196	0.31372
Data with Augmentation	10	10	0.98407	0.98431
	20	20	0.99604	0.99607
	30	30	0.99606	0.99607
Data with Feature Selection	0	0	0.46948	0.43921
Data with Feature Selection and Augmentation	10	10	0.96561	0.96470
	20	20	0.97289	0.97254
	40	40	0.95678	0.95686
Data with SHAP Optimization (10 Features)	20	20	0.97853	0.97821

Based on the simulation results, it can be observed that when no processing is applied to the statistical experimental samples, the prediction accuracy of the model is relatively low. After augmenting the volume of data within a small range, the accuracy improves; however, due to the large number of input features, the model exhibits signs of overfitting. When only feature selection is performed on the data, the accuracy of the model remains low due to insufficient data volume. By augmenting the data and simultaneously performing feature selection, the accuracy of the model improves significantly, with the prediction accuracy for accident chain length exceeding 0.95. However, as the augmentation and replication quantities increase, the model’s accuracy decreases.

To further enhance the model’s performance and interpretability, we applied SHAP for feature optimization. By analyzing the contribution of each feature using SHAP, we reduced the feature set from 15 to 10, retaining only the most influential features. This optimization not only improved the model’s accuracy but also mitigated overfitting. Specifically, the training set accuracy increased from 0.97289 to 0.97853, and the test set accuracy improved from 0.97254 to 0.97821. The SHAP-based feature optimization also enhanced the model’s interpretability by providing clear insights into the importance of each feature, enabling a more focused analysis of the factors driving domino effect accidents.

#### 4.2.3. Model robustness verification.

To verify the stability of the model and eliminate the bias caused by random division, 5-fold cross-validation was used to evaluate the optimized FC-FNN model. The dataset was randomly divided into 5 subsets. Each time, 4 subsets were used for training and 1 subset for testing, and the process was repeated 5 times. The results showed that the average accuracy of the test set was 0.977 ± 0.002, with the maximum difference between folds being 0.6% (Table 9), indicating that the model can maintain high prediction accuracy under different data distributions and effectively avoid result bias caused by accidental factors.

**Table 9:** 5 Statistical Results of 5-Fold Cross-Validation.

Fold Serial Number	Training Set Accuracy	Test Set Accuracy
Fold 1	0.979	0.976
Fold 2 Fold 3 Fold 4	0.982	0.978
	0.977	0.978
	0.975	0.973
Fold 5	0.978	0.979
Mean ± Standard Deviation	0.978 ± 0.003	0.977 ± 0.002

Note: Fluctuation Range of Test Set Accuracy = 0.006 (0.973–0.979)

Therefore, in future risk predictions of domino effect accidents, the prediction model can be utilized to determine the length of accident chains, providing a more precise construction of accident scenarios for quantitative risk assessments. This enables a better understanding of the development process and impact scope of accidents, thereby facilitating the adoption of more effective prevention and response measures.

## 5. Conclusion

This study explored domino effect accidents in chemical storage tanks, with a focus on predicting accident chain length. By analyzing 255 historical accidents from 1970 to 2024 and applying advanced machine learning techniques, several significant conclusions were drawn.

1) The chain length of domino effect accidents was precisely defined, and the dataset was categorized into atmospheric and pressurized tank accidents. The analysis revealed that pressurized storage tanks had a 69.5% secondary/higher-order accident rate, which far exceeded the 16.1% rate of atmospheric tanks. Leakage, especially in pressurized tanks (80%), was identified as the most common initiating event.
2) A fully connected feedforward neural network (FC-FNN) integrated with word embedding was developed, and input features were optimized using SHAP analysis. To address data scarcity, systematic data augmentation was implemented, significantly improving the model’s robustness. The optimized “Data with SHAP Optimization (10 Features)” model achieved an accuracy exceeding 0.978 in predicting accident chain lengths, outperforming baseline models (training/test set improvements: 0.97289 → 0.97853/ 0.97254 → 0.97821).
3) The integration of SHAP-based feature selection with an FC-FNN model establishes an interpretable framework that identifies critical factors driving accident propagation in chemical storage tanks, such as substance type and operational status. Statistical analysis further reveals that pressurized tanks are highly susceptible to multi-stage accident expansions, underscoring the urgency for targeted safety measures to mitigate domino effect risks.

To further enhance the domino effect prediction framework’s reliability and applicability, future work will focus on key aspects: Deploy IoT sensor networks to integrate real-time storage tank operational data with historical records, reducing reliance on static datasets; Use hybrid models (fusing data-driven and physics-informed networks) with embedded mechanical equations to constrain predictions by physical laws, cutting dependence on historical data; Pre – train models on public industrial datasets and fine – tune with tank – specific data to ease small – sample validation issues.
